# Advances in nowcasting influenza-like illness rates using search query logs

**DOI:** 10.1038/srep12760

**Published:** 2015-08-03

**Authors:** Vasileios Lampos, Andrew C. Miller, Steve Crossan, Christian Stefansen

**Affiliations:** 1 University College London, Department of Computer Science, London, NW1 2FD, UK; 2 Google, Flu Trends Team, London, SW1W 9TQ, UK; 3 Harvard University, School of Engineering and Applied Sciences, Cambridge, MA 02138, US

## Abstract

User-generated content can assist epidemiological surveillance in the early detection and prevalence estimation of infectious diseases, such as influenza. Google Flu Trends embodies the first public platform for transforming search queries to indications about the current state of flu in various places all over the world. However, the original model significantly mispredicted influenza-like illness rates in the US during the 2012–13 flu season. In this work, we build on the previous modeling attempt, proposing substantial improvements. Firstly, we investigate the performance of a widely used linear regularized regression solver, known as the Elastic Net. Then, we expand on this model by incorporating the queries selected by the Elastic Net into a nonlinear regression framework, based on a composite Gaussian Process. Finally, we augment the query-only predictions with an autoregressive model, injecting prior knowledge about the disease. We assess predictive performance using five consecutive flu seasons spanning from 2008 to 2013 and qualitatively explain certain shortcomings of the previous approach. Our results indicate that a nonlinear query modeling approach delivers the lowest cumulative nowcasting error, and also suggest that query information significantly improves autoregressive inferences, obtaining state-of-the-art performance.

User-generated content published on or submitted to online platforms has been the main source of information in various recent research efforts^1^,^2^,^3^. It has been shown that large data sets of social media posts and search engine queries contain signals representative of real-life patterns and are therefore indicative of social phenomena in a variety of domains, including politics^4^,^5^, finance^6^, commerce^7^,^8^, and health^9^,^10^. Focusing on health-oriented applications, early research efforts have provided empirical evidence of the informativeness of website^11^ and search engine logs^9^,^12^,^13^ for predicting influenza-like illness (ILI) rates. Google Flu Trends (GFT; http://www.google.org/flutrends)^13^, in particular, is the first real-time system to apply such methods in practice over a considerable number of countries and a large time span. Similar results were derived through the application of simple^10^,^14^ or more elaborate^15^,^16^,^17^ natural language processing techniques to content published on the micro-blogging, social networking platform of Twitter.

Data driven estimates can undoubtedly complement current sentinel surveillance systems. One of the original motivations for developing these methods is the intuition that web data could provide timely and less costly information about the prevalence of influenza in a population as opposed to traditional schemes^12^,^13^. An important distinction here is that web content can potentially access the bottom and larger part of a disease population pyramid, whereas epidemiological derivations are usually based on the subset of people that actively seek medical attention. Beyond this, places with less established health monitoring systems can greatly benefit from an adaptation of this technology.

Aside from the novelty that GFT introduced, the statistical model behind it has not been tested extensively under practical conditions. Certain works have reported on the mispredictions of GFT through an analysis of its outputs that are published online^18^,^19^,^20^. Our study proposes and compares several alternative approaches to the original GFT model based on original search query data. We explore three improvements: expanding and re-weighting the set of queries used for prediction using linear regularized regression, accounting for nonlinear relationships between the predictors and the response, and incorporating time series structure. We focus on national-level US search query data, and our task is to nowcast (i.e., to estimate the current value of)^21^,^22^ weekly ILI rates as published by the outpatient influenza-like illness surveillance network (ILINet) of the Centers for Disease Control and Prevention (CDC).

We use query and CDC data spanning across a decade (2004–2013, all inclusive) and evaluate weekly ILI nowcasts during the last five flu periods (2008–2013). The proposed nonlinear model is able to better capture the relationship between search queries and ILI rates. Given this evaluation setup, we qualitatively explain the settings under which GFT mispredicted ILI in past seasons in contrast with the improvements that the new approaches bring in. Furthermore, by combining query-based predictions and recent ILI information in an autoregressive model, we significantly improve prediction error, highlighting the utility of incorporating user-generated data into a conventional disease surveillance system.

**Modeling search queries for nowcasting disease rates**

This section focuses on supervised learning approaches for modeling the relationship between search queries and an ILI rate. We represent search queries by their weekly fraction of total search volume, i.e., for a query *q* the weekly normalized frequency is expressed by


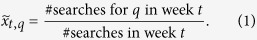


Formally, a function that relates weekly search query frequencies to ILI rates is denoted by 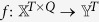, where 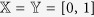 represents the space of possible query fractions and ILI percentages, *T* and *Q* are the numbers of observed weeks and queries respectively. For a certain week, 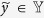 denotes the ILI rate and 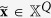 is the vector of query volumes; for a set of *T* weeks, all query volumes are represented by the *T* × *Q* matrix 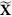. Exploratory analysis found that pairwise relationships between query rates and ILI were approximately linear in the logit space, motivating the use of this transformation across all experiments (see Supplementary Fig. S3); related work also followed the same modeling principle^13^,^23^. We, therefore, use 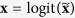 and 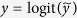, where logit(*α*) = log(*α*/(1 − *α*)), considering that the logit function operates in a point-wise manner; similarly **X** denotes the logit-transformed input matrix. We use **x**_*t*_ and *y*_*t*_ to express their values for a particular week *t*. Predictions made by the presented models undergo the inverse transformation before analysis.

**Linear models**

Previous approaches for search query modeling proposed linear functions on top of manually^9^ or automatically^13^ selected search queries. In particular, GFT’s regression model relates ILI rates (*y*) to queries via *y*_*t*_ = *β* + *w*·*z* + *ε*, where the single covariate *z* denotes the logit-transformed normalized aggregate frequency of a set of queries, *w* is a weight coefficient we aim to learn, *β* denotes the intercept term, and *ε* is independent, zero-centered noise. The set of queries is selected through a multi-step correlation analysis (see Supplementary Information [SI], *Feature selection in the GFT model*).

Recent works indicated that this basic model mispredicted ILI in several flu seasons, with significant errors happening during 2012–13^19^,^20^. Whereas various scenarios, such as media attention influencing user behavior, could explain bad predictive performance, it is also evident that the only predictor of this model (the aggregate frequency of the selected queries) could have been affected by a single spurious or divergent query. We elaborate further on this when presenting the experimental results in the following section.

A more expressive linear model directly relates individual (non-aggregated) queries to ILI. This model can be written as 
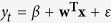
, which defines a *w*_*q*_ parameter for each of the potentially hundreds of thousands of search queries considered. With only a few hundred weeks to train on, this system is under-determined (*T* < *Q*)^24^. However, considering that most *w*_*q*_ values should be zero because many queries are irrelevant (i.e., assuming sparsity), there exist regularized regression schemes that provide solutions. One such method, known as the Lasso^25^, simultaneously performs query selection and weight learning in a linear regression setting by adding a regularization term (on **w’**s L1-norm) in the objective function of ordinary least squares. Lasso has been effective in the related task of nowcasting ILI rates using Twitter content^10^,^15^. However, it has been shown that Lasso cannot make a consistent selection of the true model, when collinear predictors are present in the data^26^. Given that the frequency time series of some of the search queries we model will be correlated, we use a more robust generalization of Lasso, known as the Elastic Net^27^. Elastic Net adds an L2-norm constraint on Lasso’s objective function and is defined by


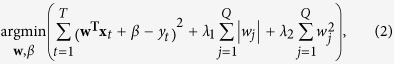


where 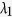, 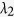 are the regularization parameters (see SI, *Parameter learning in the Elastic Net*). Compared to Lasso, Elastic Net often selects a broader set of relevant queries^24^.

**Exploring nonlinearities with Gaussian Processes**

The majority of methods for modeling infectious diseases via user-generated content are based on linear methods^10^,^13^,^14^ ignoring the presence of possible nonlinearities in the data (see Supplementary Fig. S4). Recent findings in natural language processing applications suggest that nonlinear frameworks, such as the Gaussian Processes (GPs), can improve predictive performance, especially in cases where the feature space is moderately-sized^28^,^29^. GPs are sets of random variables, any finite number of which have a multivariate Gaussian distribution^30^. In GP regression, for the inputs **x**, 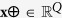 (both expressing rows of the query matrix **X**) we want to learn a function 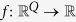 that is drawn from a 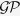 prior, *f* (**x**) ~ GP (*μ*(**x**), *k* (**x**, **x**′)), where *μ*(**x**) and *k*(**x**, **x**′) denote the mean and covariance (or kernel) functions respectively. Our models assume that *μ*(**x**) = 0 and use the Squared Exponential (SE) covariance function, defined by


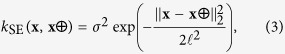


where 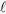 is known as the length-scale parameter and *σ*^2^ is a scaling constant that represents the overall variance. Note that 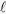 is inversely proportional to the relevancy of the feature space. Different kernels have been applied, such as the the Matérn^31^, but did not yield any performance improvements (see Supplementary Table S4). In the GP framework, predictions are conducted through 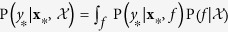, where y_*_ is the target variable, 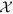 the set observations used for training, and x_*_ the current observation. Parameter learning is performed by minimizing the negative log-marginal likelihood of 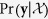, where **y** denotes the ILI rates used for training.

The proposed GP model is applied on the queries previously selected by the Elastic Net. However, instead of modeling each query separately or all queries as a whole, we first cluster queries into groups based on a similarity metric and then apply a composite GP kernel on clusters of queries. Given a partition of the search queries 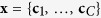, where **c**_*i*_ denotes the subset of queries clustered in group *i*, we define the GP covariance function to be


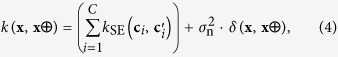


where *C* denotes the number of clusters, *k*_*SE*_ has a different set of hyperparameters (*σ*, 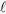) per group, and the second term of the equation models noise (*δ* being a Kronecker delta function). We extract a clustered representation of queries by applying the *k*-means++ algorithm^32^,^33^ (see SI, *Gaussian Process training details*). The distance metric of *k*-means uses the cosine similarity between time series of queries to account for the different magnitudes of the query frequencies in our data^34^. It is defined by 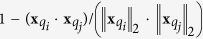, where 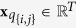 denotes a column of the input matrix **X**.

By focusing on sets of queries, the proposed method can protect an inferred model from radical changes in the frequency of single queries that are not representative of an entire cluster. For example, media hype about a disease may trigger queries expressing a general concern rather than a self-infection. These queries are expected to utilize a small subset of specific key-phrases, but not the entirety of a cluster related to flu infection. In addition, assuming that query clusters may convey different thematic ‘concepts’, related to flu, other health topics or even expressing seasonal patterns, our learning algorithm will be able to model the contribution of each of these concepts to the final prediction. From a statistical point of view, GP regression with an additive covariance function can be viewed as learning a sum of lower-dimensional functions, 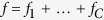, one for each cluster. As these functions have significantly smaller input space (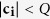, for 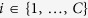), the learning task becomes much easier, requiring fewer samples and giving us more statistical traction. However, this imposes the assumption that the relationship between queries in separate clusters provides no information about ILI, which we believe is reasonable.

Denoting all ILI observations as 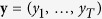, our GP regression objective is defined by the minimization of the following negative log-marginal likelihood function


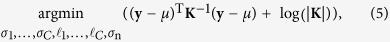


where **K** is the matrix of covariance function evaluations at all pairs of inputs, (**K**)_*i,j*_ = *k*(**x**_***i***_, **x**_***j***_), and *μ* is similarly defined as 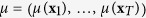. Given features from a new week, x_*_, predictions are conducted by computing the mean value of the posterior predictive distribution, *E*[y_*_|**y**, **X**, x_*_], and predictive uncertainty is estimated by the posterior predictive variance, *V*[y_*_|**y**, **X**, x_*_]^30^.

**Modeling temporal characteristics**

Autoregressive (AR) models can be used to define a more direct relationship between previously available ILI values and the current one. AR modeling has been found to improve GFT’s^20^,^35^,^36^ as well as the performance of Twitter-based systems^37^ in ILI prediction and forecasting. Due to the clear temporal correlation in the predictive errors of the query only models (see Supplementary Fig. S6), we augment our previously established query-only methods with an AR portion to gain predictive power. We do this by incorporating our prediction results into an ARMAX model, a variant of the Auto-Regressive Moving Average (ARMA) framework^38^ that generalizes simple AR models.

An ARMAX(*p*, *q*) model is often used to explain future occurrences as a function of past values, observed contemporaneous inputs, and unobserved randomness. It is composed of three parts, the AR component (*p*), the moving average component (*q*), and a regression element. At a time instance *t*, given the sequential observations 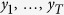, and a *D*-dimensional exogenous input **h**_*t*_, an ARMAX(*p*, *q*) model specifies the relationship


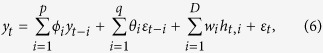


where the *ϕ*_*i*_, *θ*_*i*_, and *w*_*i*_ are coefficients to be learned and *ε*_*t*_ is mean zero Gaussian noise with some unknown variance. For fixed values of *p* and *q*, this model is trained using maximum likelihood. We extend this model with a seasonal component that incorporates yearly lags (see SI, *Seasonal ARMAX model*) and determine orders *p* and *q* as well as seasonal orders automatically by applying a step-wise procedure^39^. Instead of using all available query fractions as the exogenous input, **h**_*t*_, we only incorporate the single prediction result (*D* = 1) from a query model, 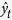. Essentially, this allows the query-only model to distill all of the information that search data have to offer about the ILI rate at time *t*, before using this meta-information in the ARMAX procedure. Predictive intervals are estimated for each autoregressive nowcast through the maximum likelihood variance of the model.

## Results

We evaluate our methodology on held out ILI rates and normalized query frequencies from five consecutive periods matching the influenza seasons from 2008 to 2013, as defined by CDC (see SI, *Materials* and Supplementary Fig. S1). For each test period (flu season *i*), we train a model using all previous data points (dating back to January, 2004, i.e., from the first flu season in our data to season *i* − 1); this holds for the ARMAX models as well with the only difference that training starts from season 2008–09 (to include out-of-sample ILI rate inferences in the AR training process). We only maintain search queries that exhibit a Pearson correlation of ≥.5 with the ILI rates in the training data. In this way, we reduce the possibility of learning models that overfit by incorporating unrelated queries, and also eliminate negatively correlated content under the assumption that for our specific task anti-correlation is often due to seasonal patterns (e.g., queries seeking treatment for snake bites) rather than causal links. We note that in order to establish a consistent comparison, the GFT estimates in the paper have been re-computed based on the query data set used in our experiments as well as our evaluation scenario; therefore, there might exist differences compared to the GFT web platform’s outputs. The GP model uses a fixed number of *k* = 10 clusters (see Supplementary Table S3 for experiments with different cluster sizes). Performance is measured by using the following metrics between inferred and target ILI values: Pearson correlation *r* (which is not always indicative of the magnitude of error), Mean Absolute Error (MAE) and Mean Absolute Percentage of Error (MAPE) (defined in SI, *Performance metrics*).

### Query-only models

**Figure 1:**
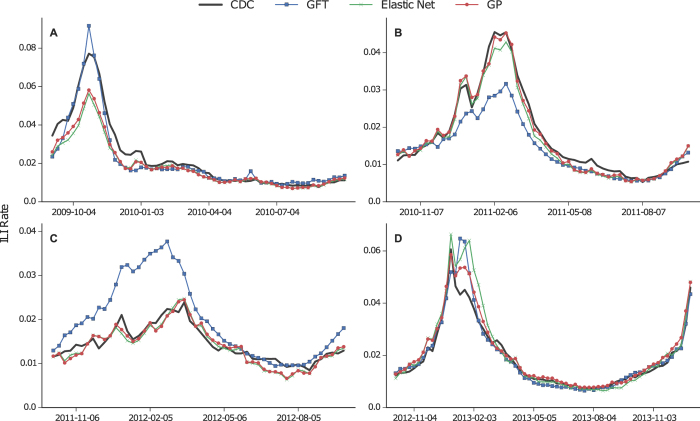
Graphical comparison between ILI nowcasts based on query-only models and the ILI rates published by CDC. (**A–D**): Flu seasons 2009–10, 2010–11, 2011–12 and 2012–13 respectively.

Table 1 enumerates the performance results for the three query-only models (GFT, Elastic Net and GP) and Fig. 1 presents the respective graphical comparison between predicted and actual ILI rates (Supplementary Fig. S2 shows the results for 2008–09). Further details, such as the number of selected or nonzero weighted queries per case and model are shown in Supplementary Table S2. Evidently, the GP model outperforms both GFT and Elastic Net models. Using an aggregation of all inferences and the MAPE loss function, we see that Elastic Net yields an absolute performance improvement of 8.5% (relative improvement of 41.7%) in comparison to GFT. The GP model in comparison to Elastic Net improves predictions further by 1.1% (relative improvement of 9.2%). We also observe that both Elastic Net and GP models cannot capture the ILI rate during the peak of the flu season for 2009–10, whereas the GFT model over-predicts it. This could be a consequence of the the fact that 2009–10 was a unique flu period, as it is the only set of points expressing a pandemic in our data (H1N1 swine flu pandemic).

By measuring the influence of individual queries or clusters in each nowcast, we conduct a qualitative evaluation of the models, aiming to interpret some prediction errors. Our influence metric computes the contribution of a query or a cluster of queries by comparing a normal prediction outcome with an output had this query or cluster been absent from the input data (see SI, *Estimation of query and cluster influence in nowcasts*). The GFT model is very unstable across the different flu seasons, sometimes exhibiting the smallest error (season 2009–10), and other times severely mispredicting ILI rates (seasons 2008–09, 2010–11 and 2011–12). Through an examination of a 21-week period (04/12/2011 to 28/04/2012), where major over-predictions occur (see Fig. 1C), and the estimation of the percentage of influence for each query in the weekly predictions, we deduced that queries unrelated to influenza were responsible for major portions of the final prediction. The query ‘rsv’ (where RSV stands for Respiratory Syncytial Virus) accounts on average for 24.5% of the signal, overtaking the only clearly flu-related query with a significant representation (‘flu symptoms’ expressing 17.5% of the signal); the top five most influential queries also include ‘benzonatate’ (6.2%), ‘symptoms of pneumonia’ (6%) and ‘upper respiratory infection’ (3.9%), all of which are either not related to or may have an ambiguous contribution to ILI. Hence, the predictions were primarily influenced by content related to other types of diseases or generic concern, something that resulted in an over-prediction of ILI rates. For the same 21-week period, we performed a similar analysis on the features from the significantly better performing Elastic Net model. Firstly, the influence of each query is less concentrated, something expected given the increased number of nonzero weighted queries forming up the model (316 queries in Elastic Net vs. 66 in GFT). The features with the largest contribution were ‘ear thermometer’ (3.1%), ‘musinex’ (2.4%)—a misspelling of the ‘mucinex’ medicine, ‘how to break a fever’ (2.2%), ‘flu like symptoms’ (2.1%) and ‘fever reducer’ (2%), all of which may have direct or indirect connections to ILI. Note that none of the top five GFT features received a nonzero weight by Elastic Net, hinting that the latter model provided a probably better feature selection in this specific case.

During the last testing period (2012–13), we observe that the Elastic Net model marginally over-predicts the peak ILI rate, and sustains the same behavior in the 7 weeks that follow (Fig. 1D, weeks from 23-12-2012 to 16-02-2013). In the same 8-week period, the GP model manages to reduce Elastic Net’s MAPE from 31.9% to 14.9% (53% reduction of error). Elastic Net’s top influential queries for that time interval include irrelevant entries. Some examples, together with their average influence percentage and ranking, are ‘muscle building supplements’ (1.7%, 4th), ‘cold feet’ (1.3%, 10th), ‘what is carbon monoxide’ (1.2%, 13th) and ‘chemical formula for sugar’ (0.9%, 20th). The most influential query is ‘fever reducer’ (2.4%), a wording focused on a symptom of flu, but also of other diseases. We now determine the influence of each query cluster in the final GP prediction. Interestingly, the preceding queries, which may be unrelated to ILI (including ‘fever reducer’), are members of clusters with a very small average influence (from < .01% to a maximum of 1.3%). Notably, the most influential cluster includes queries about the ‘nba injury report’ (62.3%), whereas the second is clearly about flu (31.5%; ‘flu symptoms’, ‘flu prevention’ and ‘flu or cold’ were the most central terms). Initially, the model’s former choice may seem peculiar, however, the time series of ‘nba injury report’ reveal that this query is a great indicator of the winter season (see Supplementary Fig. S7). Nevertheless, *k*-means has separated this cluster from the disease-oriented ones.

In order to understand the nature and influence of the clustering in the GP models we draw our focus on the first testing period (2008–09; see Supplementary Fig. S2). There, the cluster with the strongest influence in the predictions (85.8% on average) is formed by queries that are very closely related to flu. The top five ones in terms of cluster centrality are the misspelled ‘flu symptons’, followed by ‘flu outbreak’, ‘how to treat the flu’, ‘flu season’ and ‘flu vs cold’. The second cluster has a significantly smaller influence (3.3%) and contains generic queries that seek information about various health conditions; the most central queries are ‘causes of down syndrome’, ‘trench foot’, ‘what causes pneumonia’, ‘what is a genotype’ and ‘marfan syndrome pictures’. Looking at the remaining clusters, all of which have a moderate degree of influence, we see that their contents reflect different types of diseases or seasonal patterns. In particular, the top-3 most central queries in the third (2.6%), fourth (2.4%) and fifth (1.6%) clusters in terms of predictive influence are respectively {‘croup in infants’, ‘wooping cough’, ‘pnemonia symptoms’}, {‘best decongestant’, ‘dogs eating chocolate’, ‘how to stop vomiting’} and {‘dr king’, ‘rsv in adults’, ‘charles drew’}. Finally, there exists one more cluster that covers the topic of flu, but has a minor influence (1.3%). Notably, the queries of that cluster are less likely to have been issued by people with ILI as they are looking for more generic information about the disease, e.g., ‘flu duration’, ‘influenza b’, ‘cdc flu map’, ‘type a influenza’ and ‘tamiflu side effects’ are the most central queries.

### Query models in an AR process

**Figure 2:**
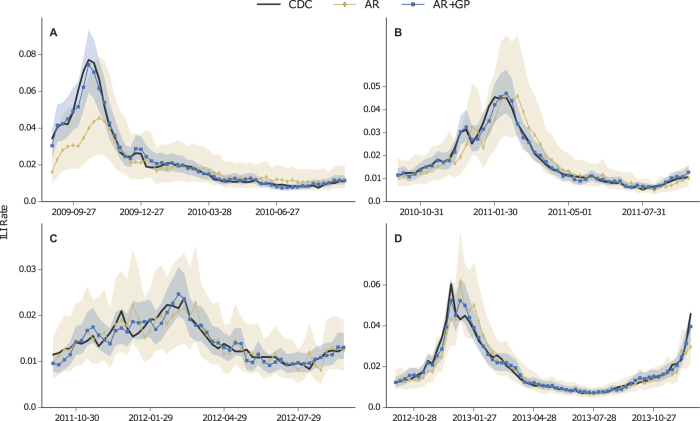
Comparison of nowcasts between an autoregressive baseline model which is based only on ILI data (AR) and the AR + GP model. In both occasions the lag is set to 2 weeks and the corresponding uncertainty intervals are highlighted. (**A–D**): Flu seasons 2009–10, 2010–11, 2011–12 and 2012–13 respectively.

**Figure 3:**
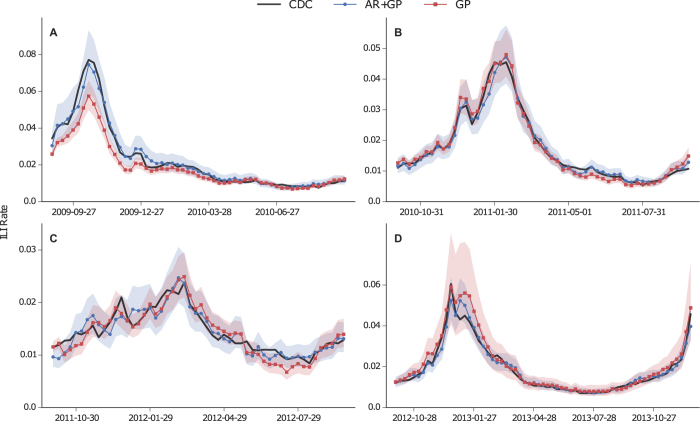
Comparison of nowcasts between the autoregressive model AR + GP and the query-only GP model together with their corresponding uncertainty intervals. (**A–D**): Flu seasons 2009–10, 2010–11, 2011–12 and 2012–13 respectively.

Nowcasts from the GFT, Elastic Net and GP models are used as a one-dimensional exogenous input in the ARMAX model defined by Eq. (6). We also include an AR baseline prediction, where only CDC data are used. During training, we compare multiple orders (values for *p* and *q*) and select a model based on Akaike Information Criterion^39^. AR model training starts from season 2008–09 (to be able to include out-of-sample query only predictions), and the first period of testing is 2009–10. Table 2 enumerates the performance results of the applied ARMAX models, using CDC ILI data with a time lag of 1 or 2 weeks from the current prediction. The best performance is achieved when GP nowcasts are used in the ARMAX framework (AR + GP), with a cumulative MAPE equal to 5.7% or 7.3% when a 1- or 2-week lag is applied respectively. Focusing on the 2-week lag as it reflects the delay of the actual CDC ILI reporting, the AR + GP model yields a 5.2% improvement over AR + Elastic Net, 28.4% over AR + GFT and 49% over the AR ILI baseline. Figure 2 plots the AR + GP nowcasts against the baseline AR and the ground truth ILI rates, when a 2-week lag is assumed. Apart from the significant prediction accuracy that the AR + GP model provides, we also observe that the incorporation of query information dramatically reduces the uncertainty of the predictions under all testing periods. Figure 3 draws an additional comparison, where the query-only model (GP) is plotted against its AR version. Interestingly, for the testing period 2009–10, it becomes evident that the AR + GP model is now capturing the peak of the flu season. Furthermore, the prediction intervals become tighter, especially when ILI rates are high (see Fig. 3D). It is important, however, to note that the uncertainty in the query-only prediction is not propagated through to prediction; the AR + GP procedure sees the GP regression as a form of data preprocessing.

We found that predictive errors in query-only models display auto-correlated structure that can be exploited for improved prediction (see Supplementary Fig. S6). The contribution of the ARMAX framework is that it can directly model this, effectively resetting the mean value of the prediction to a more likely location. An examination of the predictive period and the Q—Q plot of normalized logit-space errors (see Supplementary Fig. S5), shows a systematic bias in query-only experiments that is mitigated by the addition of the AR components. The improvement of the AR + GP and AR + Elastic Net models over the AR + GFT can be attributed to the higher query-only correlation with the CDC ILI signal, and the AR component’s ability to incorporate information about the natural autocorrelation in the signal.

A more fine-grained analysis of the predictions, when they really matter, i.e., during the peaking moments of a flu season, provides additional support for the improvements brought by the new query modeling methodology. Including weeks that belong to the .85 quantile of the seasonal CDC ILI rates (7 to 10 weeks per season), we measure the nowcasting performance of all the investigated models; the results are enumerated in Table 3. There, we observe that the GP model exhibits a similar MAPE to its general average performance, whereas the other models are much more error prone. For example, in the query-only results, Elastic Net’s cumulative MAPE during peak flu periods increases to 15.8% (from 11.9% overall), whereas GP’s error rate remains at the same levels (11% from 10.8%).

## Discussion

We have presented an extensive analysis on the task of nowcasting CDC ILI rates based on queries submitted to an Internet search engine. Previously proposed (GFT) or well established (Elastic Net) methods have been rigorously assessed, and a new nonlinear approach driven by GPs has been proposed. In addition, query-only models were complemented by autoregressive components, merging traditional syndromic surveillance outputs with inferences based on user-generated content. Overall the nonlinear GP approach, either in a query-only or an autoregressive format, performed better than the investigated alternatives. By conducting a qualitative analysis, we attempted to understand the shortcomings or the benefits of these regression models. A clear disadvantage of the original GFT model^13^ was the merging of all query data into a single variable; in several occasions, this aggregation was injecting non directly flu-related queries (referring to a different disease or expressing a generic concern) that significantly affected the final prediction. Such queries may have been removed through manual inspection, making the whole system semi-automatic, but nevertheless this would not have resolved the overfitting caused by the limited expressiveness of the learning algorithm.

It is important to note that the presented AR + GP model achieves state-of-the-art performance compared to related recent works in the literature. In particular, the AR model proposed by Paul and Dredze^37^ that combines Twitter-based estimates^16^,^40^ with prior CDC ILI rates, reaches a MAE (×10^2^) of .190 when a 1-week lagged ILI rate is incorporated. Experiments in this paper were conducted for the years 2011–14; our AR + GP model (with a 1-week lag) yields a cumulative MAE of .098 during 2011–13, whereas the MAE of the GP query-only model is .156. Similarly, our model outperforms AR setups built specifically on top of the publicly available GFT outputs, namely from Preis and Moat^36^, with a MAE of .133 (during 2010–13), and from Lazer *et al.*^20^, with a MAE of .232 (during 2011–12).

Confirming the estimations of related work^7^,^20^, the previous GFT query-only method did not have a stronger predictive power than a 2-week delayed AR model based on CDC reports (20.4% vs. 14.3% MAPE, respectively). However, by allowing a greater expressiveness and by performing regularized regression some of the modeling issues were resolved. In fact, the query-only model based on Elastic Net regularization delivered a much better performance (11.9% MAPE), which improved with the GP model (10.8% MAPE). The operation on clusters of related queries makes the proposed GP model more robust to sudden changes in the frequency of single queries, that may happen due to media hype or other data corruption scenarios (e.g., ‘fake’ query attacks).

Looking further into the AR modeling and judging via the empirical predictive performance, we deduce that CDC data can be a useful addition when their lag is up to 4 or 5 weeks (see Supplementary Table S5). After this point, the AR + GP model does not benefit from the addition of ILI information, i.e., its performance falls back to the levels of query-only modeling. However, without query information, the equivalent AR CDC-based projections from a 3-week lag and onwards are becoming quite unreliable. This highlights some additional potential use cases of search query based surveillance, for example, in situations where a health surveillance system is blocked for a period greater than 2 weeks or ILI estimates are released on a monthly basis.

An interesting conclusion which came of as an intermediate result of our analysis was the observation that basic natural language processing techniques, such as stemming, stop-word removal or the extraction of n-grams from the search query text, did not improve performance (see Supplementary Table S1). This can potentially highlight the quality of the signal enclosed in the data, at least at a national level density, as text preprocessing worsens rather than enhances information.

We note that a generic limitation for this line of research is the non-existence of a solid ‘gold standard’. Traditional health surveillance data is based on the subset of the population that uses healthcare services, and we are aware that on average non-adults or the elderly are responsible for the majority of doctor visits or hospital admissions^41^. Thus, the provided ILI rates may not always form a definite ground truth. Those potential biases are carried onto the query-only models through the means of supervised learning, and their impact becomes stronger in the updates of an AR model. On a more technical level, the increased expressiveness of the nonlinear GP model also comes at a price of interpretability, making harder to isolate the contribution of a single query. However, we can still interrogate the hyperparameters of each query cluster and see which contributes most to the marginal variance of predictions.

As these findings are turned into a real-time system, some additional and equally important concepts need to be investigated, such the optimal length of the training window, i.e., how and when should the system forget past information in order to adapt better to newly formed concepts. Research on combining the multiple user-generated resources that have emerged in the recent years needs to be attempted, hypothesizing that it may provide a greater penetration in the population and, consequently, an even better accuracy. Finally, extensions of this work should consider combining the presented core models with network-based, more epidemiology-centric findings, where interesting properties of an infectious disease, such as geography^42^ or the source of spreading^43^,^44^, could be potentially captured.

## Additional Information

**How to cite this article**: Lampos, V. *et al.* Advances in nowcasting influenza-like illness rates using search query logs. *Sci. Rep.* **5**, 12760; doi: 10.1038/srep12760 (2015).

## Supplementary Material

Supplementary Information

## Figures and Tables

**Table 1:** Performance of all the investigated query-only models in nowcasting ILI rates using Pearson correlation (*r*), MAE, and MAPE between predictions and response data across the five identified flu periods.

Period	Weeks	**GFT**			**Elastic Net**			**GP**		
		***r***	**MAE × 10^2^**	**MAPE(%)**	***r***	**MAE × 10^2^**	**MAPE(%)**	***r***	**MAE × 10^2^**	**MAPE(%)**
2008–09	48	0.66	0.490	30.8	0.94	0.180	10.6	0.94	0.175	10.6
2009–10	57	0.97	0.324	14.4	0.99	0.499	15.1	0.99	0.451	14.6
2010–11	52	0.97	0.390	18.0	0.99	0.168	11.3	0.99	0.130	9.5
2011–12	52	0.92	0.550	33.1	0.94	0.131	9.8	0.94	0.129	9.8
2012–13	65	0.96	0.209	9.5	0.98	0.286	12.1	0.99	0.199	9.4
2008–13	274	0.89	0.381	20.4	0.92	0.260	11.9	0.95	0.221	10.8

**Table 2:** Nowcasting performance (*r*, MAE × 10^2^, MAPE(%)) of autoregressive ILI estimators (AR + Query-model) implemented by incorporating query-only nowcasts and lagged ILI rates from CDC into an ARMAX model.

**Period(Lag)**	**AR**			**AR + GFT**			**AR + Elastic Net**			**AR + GP**		
	***r***	**MAE**	**MAPE**	***r***	**MAE**	**MAPE**	***r***	**MAE**	**MAPE**	***r***	**MAE**	**MAPE**
2009–10 (1)	0.98	0.314	11.2	0.99	0.173	7.4	≈1	0.110	5.1	≈1	0.123	5.9
2010–11 (1)	0.98	0.163	8.6	0.98	0.150	7.9	0.99	0.085	5.3	0.99	0.084	5.2
2011–12 (1)	0.95	0.092	6.4	0.95	0.098	6.8	0.95	0.099	6.9	0.95	0.101	7.0
2012–13 (1)	0.97	0.170	6.7	0.98	0.148	6.2	0.99	0.135	6.6	0.99	0.108	5.0
2009–13 (1)	0.97	0.187	8.2	0.98	0.143	7.0	0.99	0.109	6.0	0.99	0.105	5.7
2009–10 (2)	0.92	0.605	21.4	0.97	0.274	10.7	0.99	0.146	6.6	0.99	0.163	7.6
2010–11 (2)	0.92	0.305	15.6	0.95	0.250	12.5	0.99	0.110	6.7	0.99	0.102	6.3
2011–12 (2)	0.88	0.141	9.9	0.88	0.143	9.8	0.93	0.120	8.4	0.93	0.124	8.6
2012–13 (2)	0.92	0.280	10.6	0.95	0.206	8.3	0.98	0.187	8.7	0.98	0.146	7.0
2009–13 (2)	0.87	0.336	14.3	0.96	0.219	10.2	0.99	0.144	7.7	0.99	0.135	7.3

AR columns provide baselines, where only CDC ILI rates are used, and ‘Lag’ specifies the number of weeks (1 or 2) separating a nowcast and the latest ILI input from CDC.

**Table 3:** MAPE(%) of query-only and autoregressive (2-week lag) nowcasts during the peaking weeks of each flu period.

Period	#of weeks	*θ*	MAPE					
			GFT	ElasticNet	GP	AR + GFT	AR + ElasticNet	AR + GP
2008–09	7	2.47%	17.3	4.3	3.7	—	—	—
2009–10	9	4.21%	7.8	29.9	24.7	10.9	5.7	6.3
2010–11	8	3.11%	30.7	6.9	4.3	15.4	6.5	6.0
2011–12	8	1.93%	55.2	8.4	7.2	9.4	7.6	7.3
2012–13	10	3.26%	16.1	24.0	12.3	15.7	12.7	9.9
2008(9)–13	42 (35)	NA	24.8	15.8	11.0	13.0	8.3	7.5

Peaking weeks are defined by the 0.85 quantile (≥*θ*) of the CDC ILI rates per season.
