# Supplementary material for: Advances in nowcasting influenza-like illness rates using search query logs
Source: Sci Rep. 2015 Aug 3;5:12760. doi: 10.1038/srep12760 (PMC4522652; doi:10.1038/srep12760)
Supplement: Supplementary Information [file srep12760-s1.pdf]

Supplementary Information for

Advances in nowcasting influenza-like illness rates using search query logs

Vasileios Lampos[1,2,*], **Andrew C. Miller**[2,3], **Steve Crossan**[2] and **Christian Stefansen**[2]
1. University College London, Department of Computer Science, London, NW1 2FD, UK
2. Google, Flu Trends Team, London, SW1W 9TQ, UK
3. Harvard University, School of Engineering and Applied Sciences, Cambridge, MA 02138, US
* v.lampos@ucl.ac.uk

Materials

Search query data

For our analysis, we have used a volume of millions (issued) search queries geo-located in the US, dated from 04/01/2004 to 28/12/2013 (521 weeks) and filtered by Google's embedded health vertical classifier. This is a relaxed topic classifier, and as a result many search queries are not directly related to the topic of health. The data have been anonymized and aggregated before conducting experiments. By performing an intersection among frequently occurring search queries geo-located in the 10 US regions, we ended up with the weekly frequencies of 49,708 queries (from an original set of 297,057 queries).

Flu seasons

The search query data used in our experiments are spread across 10 years and encompass 9 complete and 2 partial flu seasons (as identified by CDC). We test the performance of the proposed ILI models on data from the latest flu seasons. These were 2008-09 (48 weeks, 28/9/2008 to 29/8/2009), 2009-10 (57 weeks, 30/8/2009 to 2/10/2010), 2010-11 (52 weeks, 3/10/2010 to 1/10/2011), 2011-12 (52 weeks, 2/10/2011 to 29/9/2012) and 2012-13 (65 weeks, 30/9/2012 to 28/12/2013).

Official health reports

The CDC operates an outpatient ILI surveillance network (ILINet) consisting of more than 2,900 healthcare providers in all US states. According to the CDC[1] "ILI is defined as fever (temperature of 100°F [37.8°C] or greater) and a cough and/or a sore throat without a known cause other than influenza." ILI rates are published on a weekly basis (usually lagged by a 2-week window) indicating the percentage of ILI prevalence at a national level. We use ILINet's rates in the aforementioned 521 weeks (see Fig. S1) to train and evaluate our models throughout our work.

Performance metrics

Given a set $y = y_1, \ldots, y_N$ of ground truth values and $\hat{y} = \hat{y_1}, \ldots, \hat{y_N}$ of predictions, we apply the following metrics to assess predictive performance:

- Pearson correlation (r), defined by

$$r = \frac{1}{N-1}\sum_{t=1}^{N}\left(\frac{y_t - \mu(y)}{\sigma(y)}\right)\left(\frac{\hat{y}_t - \mu(\hat{y})}{\sigma(\hat{y})}\right), \tag{1}$$

  where $\mu(\cdot)$ and $\sigma(\cdot)$ are the sample mean and standard deviation respectively.

- Mean Absolute Error (MAE), defined by

$$\text{MAE}(\hat{\mathbf{y}}, \mathbf{y}) = \frac{1}{N}\sum_{t=1}^{N}|\hat{y}_t - y_t|. \tag{2}$$

- Mean Absolute Percentage of Error (MAPE), defined by

$$\text{MAPE}(\hat{\mathbf{y}}, \mathbf{y}) = \frac{1}{N}\sum_{t=1}^{N}\left|\frac{\hat{y}_t - y_t}{y_t}\right|. \tag{3}$$

We note that performance is measured after reversing the logit transformation (see below).

Why is the logit transformation appropriate?

The logit transformation takes the original signal in the range (0,1) and transforms it to $(-\infty, \infty)$. Exploratory analysis (see Fig. S3) found that pairwise relationships between query rates and ILI were approximately linear in the logit space, motivating the use of the transformation across all experiments. In fact, Elastic Net's[2] performance significantly improves, when applied on logit-transformed query frequencies (see Table S1).

Model training details

We restate that the our input information is the logit-transformed, normalized frequency time series of search queries, $\mathbf{X} \in \mathbb{R}^{T \times Q}$, where T and Q denote the number of weeks and queries respectively. In each period of the testing process the input queries are the ones with $r \geq .5$ Pearson correlation with the corresponding ILI data (in the training data points). We denote them as $\mathbf{Z} \in \mathbb{R}^{T \times Q'}$, where $Q' \leq Q$. A consecutive in time set of queries, $\mathbf{Z}' \in \mathbb{R}^{T' \times Q'}$, representing the following flu season is used for testing.

Feature selection in the GFT model

Following the feature selection method of the original paper to the extent possible,[3] we select queries by performing the following steps:

1. Training data ($\mathbf{Z}$) are divided into two chronological subsets, one containing points from the latest flu season (denoted by $\mathcal{Z}_{\mathrm{v}}$) and the other containing the points (weeks) prior to the latest season ($\mathcal{Z}_{\mathrm{t}}$).
2. Using each query present in $\mathbf{Z}$ as input, we train a GFT model (on logit-transformed time series) on $\mathcal{Z}_{\mathrm{t}}$ and test its performance on $\mathcal{Z}_{\mathrm{v}}$.
3. Based on the MAE loss function we identify the 150 best performing queries.
4. Starting from the best performing query and adding up one query (the next best in performance) each time, we train a GFT model based on $\mathcal{Z}_{\mathrm{t}}$ and measure its performance on data from $\mathcal{Z}_{\mathrm{v}}$ using the Pearson correlation metric.
5. We select the set of queries that exhibits the best correlation.

Parameter learning in the Elastic Net

To reduce the degrees of freedom and to create a balanced regularization between the L1 and L2 norms of the Elastic Net, we have set $\lambda_1 = 2\lambda_2$, i.e., in the relationship between the two regularizers, i.e., $\lambda_2 = \lambda_1(1-\alpha)/2\alpha$, we have set $\alpha = .5$ (see paper's Eq. 2). We learn Elastic Net's parameters (weights and λ_1) as follows:

1. Training data ($\mathbf{Z}$) are divided into N chronological subsets, each one containing points from one of the N flu seasons covered by the data.
2. An Elastic Net model is fitted on all-but-one subsets of the training set, and 10^3 λ_1 candidates are tested for their performance on the held-out subset using MAPE.
3. The best performing λ_1 is used to train a model (learn the weight for each query) based on the entirety of the training data.

Elastic Net is implemented using MATLAB®'s built-in `lasso` function, with non default settings, disabling standardization and setting the alpha parameter to .5.

Gaussian Process training details

To optimize the initialization of the model's hyperparameters (see paper's Eq. 5), we perform a series of random hyperparameter initializations, run a few iterations (10 to 20) of the inference process, and then select the hyperparameter initialization that produces the lowest negative log-marginal likelihood.[4]

The GP model described in the main paper uses the squared exponential (SE) kernel (k_{SE}) as the building block for its covariance function. A different kernel (the Matérn with $\nu = 5/2$[5]) was also applied for completeness. Table S4 compares the nowcasting performance between these two kernels. Note that the SE kernel performs slightly better than the Matérn.

As specified in the main text, we fix the number of k-means clusters to 10. Table S3 enumerates performance results for different cluster sizes. When k-means clustering is applied, we perform 50 randomly initialized runs in order to reduce the centroid initialization bias.

GP model design, parameter learning and inference are conducted using the Gaussian Processes for Machine Learning toolbox,[6] whereas for k-means we used the built-in MATLAB® function (`kmeans`).

Seasonal ARMAX model

A seasonality component in the ARMAX function incorporates further information into the model. In all of our experiments, the length of the season is fixed to 52 weeks (1-year long). The full model description, which extends Eq. 6 in the main paper, becomes

$$y_t = \underbrace{\sum_{i=1}^{p} \phi_i y_{t-i} + \sum_{i=1}^{J} \omega_i y_{t-52-i}}_{\text{AR and seasonal AR}} + \underbrace{\sum_{i=1}^{q} \theta_i \epsilon_{t-i} + \sum_{i=1}^{K} \nu_i \epsilon_{t-52-i}}_{\text{MA and seasonal MA}} + \underbrace{\sum_{i=1}^{D} w_i h_{t,i}}_{\text{regression}} + \epsilon_t \,, \tag{4}$$

where ω_i and ν_i are lagged variable parameters of order J and K, respectively. We estimate a series of models using[7] and choose the model that minimizes the Akaike Information Criterion (AIC);[8] therefore all the hyper-parameters are automatically determined. By observing these parameters, the evidence of seasonality in the signal is far less clear in the first prediction period (when there are fewer samples from previous years) than the evidence in the last prediction period (where there are examples of many preceding seasons). More precisely, the first few prediction periods do not incorporate a yearly lag, whereas as the last two tend to incorporate an AR seasonal lag of order 1 and a moving average seasonal lag of order 1.

Furthermore, the estimation procedure includes a search over integrated components, augmenting the ARMAX to the autoregressive integrated moving average regression (ARIMA). The integrated part of the model refers to an initial differencing step aimed at removing non-stationarities present in the time series. As more evidence of stationarity is presented in later prediction periods, the inference procedure settles on no integrated effect, as seen in the outputs listed in Fig. S8 and S9.

Query text preprocessing

To assess whether standard text preprocessing can improve the prediction performance, we preprocessed the original data and created the following outputs:

1. ***n*-grams**: We n-grammed queries extracting 1- to 4-grams (an n-gram is a set of n words or text tokens). We maintained the n-grams with > 5 daily average occurrences. This resulted in a set of 79,872 n-grams.

2. **Preprocessed queries**: We removed a set of 536 common English stop-words from each query, stemmed (using Porter's algorithm[9]) the 1-grams in the queries when they were two or more characters long, removed 1-grams that were one character long, and finally deduplicated the remaining queries (queries that ended up being the same were merged into one variable). This preprocessing reduced the number of queries to 42,708 (7,000 fewer than the original data set).

The performance of Elastic Net under these two preprocessed data sets is enumerated in Table S1. We conclude that when Elastic Net is applied on the raw (non preprocessed) data, it performs better on average.

Estimation of query and cluster influence in nowcasts

For the linear methods (GFT and Elastic Net), we apply the following approach to distill the influence of single queries in a prediction:

- A query q_i is removed from the feature space.
- Nowcasts for ILI are computed (excluding q_i).
- The absolute relative difference (%) between the nowcasts with and without q_i is measured.
- By normalizing this percentage across all queries (so that for each query-nowcast pair it is from 0 to 1), and then averaging (per query) for a period of N weeks, we extract the average influence of that query in the nowcasts during these N weeks.

It is hard to investigate single query influence in the GP model. However, taking advantage of the additive decomposition of the kernels (each applied on a different cluster), we can measure the influence of each cluster. In the GP model, a nowcast is

computed by

$$
\begin{aligned}
\hat{y}_t &= k_{\mathrm{SE}}(\mathbf{x}_t, \mathbf{X}) \left(\mathbf{K} + \sigma_n^2 \mathbf{I}\right)^{-1} \mathbf{y} \\
&= \left(\sum_{c=1}^{C} k_{\mathrm{SE}}(\mathbf{x}_{t,c}, \mathbf{X}_c) \right) \left(\mathbf{K} + \sigma_n^2 \mathbf{I}\right)^{-1} \mathbf{y} \\
&= \sum_{c=1}^{C} k_{\mathrm{SE}}(\mathbf{x}_{t,c}, \mathbf{X}_c) \left(\mathbf{K} + \sigma_n^2 \mathbf{I}\right)^{-1} \mathbf{y} \,,
\end{aligned}
\tag{5}
$$

where $\mathbf{x}_c$ denotes the inputs for a cluster c. This equation breaks down into an additive decomposition of C terms, each one only relying on the terms of a cluster. Therefore, similarly to the linear models, we can exclude one cluster, compute a nowcast, and then compute its influence by comparing it to the actual prediction.

References

1. Centers for Disease Control and Prevention. Case definitions for infectious conditions under public health surveillance. Morbidity and Mortality Weekly Report 46 (1997). URL http://www.cdc.gov/mmwr/preview/mmwrhtml/00047449.htm. Accessed 11.06.2015.
2. Zou, H. & Hastie, T. Regularization and variable selection via the elastic net. *J Roy Stat Soc B Met* **67**, 301–320 (2005).
3. Ginsberg, J. *et al.* Detecting influenza epidemics using search engine query data. *Nature* **457**, 1012–1014 (2009).
4. Turner, R. D. *Gaussian Processes for State Space Models and Change Point Detection*. Ph.D. Thesis, University of Cambridge (2011).
5. Matérn, B. *Spatial Variation* (Springer, 1986).
6. Rasmussen, C. E. & Nickisch, H. Gaussian Processes for Machine Learning (GPML) toolbox. *J Mach Learn Res* **11**, 3011–3015 (2010).
7. Hyndman, R. J. & Khandakar, Y. Automatic Time Series Forecasting: The forecast Package for R. *J Stat Softw* **27**, 1–22 (2008).
8. Sakamoto, Y., Ishiguro, M. & Kitagawa, G. *Akaike Information Criterion Statistics* (Springer, 1986).
9. Porter, M. F. An algorithm for suffix stripping. *Program* **14**, 130–137 (1980).

Model	r	MAE$\times 10^2$	MAPE (%)
Elastic Net	.92	.260	11.9
Elastic Net (no logit)	.75	.511	25.7
Elastic Net (n-grams)	.93	.304	15.7
Elastic Net (preprocessed queries)	.94	.265	13

Table S1. Cumulative performance (2008-2013) for Elastic Net models without the logit transformation or when text preprocessing is applied. When logit transformation is applied, performance is measured after inverting logit-space estimates.

Period	$r \geq .5$	GFT	Elastic Net
2008–09	1,477	92	196
2009–10	1,029	8	241
2010–11	782	66	277
2011–12	764	59	316
2012–13	632	3	360

Table S2. Number of features in the various phases of the training process, showing how many queries are above the $r \geq .5$ threshold, and the subset of them selected by either GFT or Elastic Net.

Number of clusters	r	MAE$\times 10^2$	MAPE (%)
1	.91	.273	12.3
2	.92	.266	12.2
4	.93	.243	11.4
6	.92	.246	11.6
8	.94	.236	11.7
10	.95	.221	10.8
12	.94	.234	11.2

Table S3. Cumulative performance (2008-2013) of GP model with various numbers of clusters.

Covariance function	r	MAE$\times 10^2$	MAPE (%)
SE	.95	.221	10.8
Matérn	.95	.228	11

Table S4. Performance comparison of the optimal GP model (10 clusters) when a different covariance function (Matérn) is used.

		**Lag (number of weeks)**			
		3	4	5	6
r	**AR**	.77	.66	.56	.47
	**AR+GP**	.98	.98	.98	.98
MAE	**AR**	.444	.544	.635	.709
	**AR+GP**	.160	.183	.195	.205
MAPE	**AR**	19	23.8	28.3	32.4
	**AR+GP**	8.7	10.1	10.9	11.5

Table S5. Cumulative Pearson correlation (r), MAE$\times 10^2$ and MAPE(%) across four prediction periods (from 2009 to 2013) for lags equal up to 6 weeks (see Table 2 for results on 1 and 2-week lags). To assist interpretation, a 4-week lagged prediction ($\hat{y}_t$) uses the present search query prediction, but relies only on CDC ILI values up to y_{t-4}.

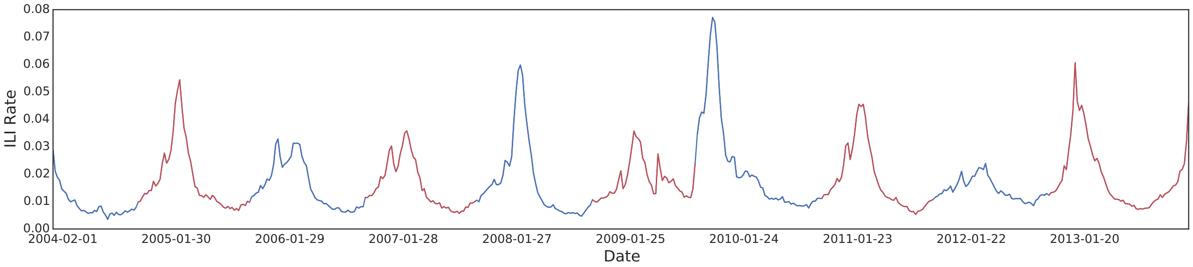

Figure S1. CDC ILI rates for the US covering 2004 to 2013, i.e., the time span of the data used in our experimental process. Flu periods are distinguished by color.

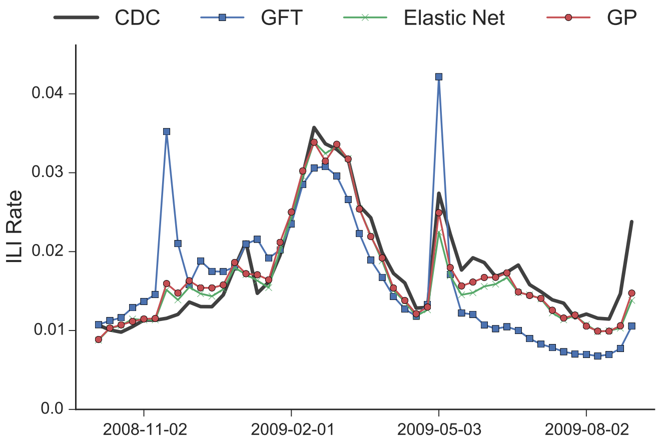

Figure S2. Comparison of query-only predictions for all investigated models during the flu season 2008-09 (omitted from main text for space reasons).

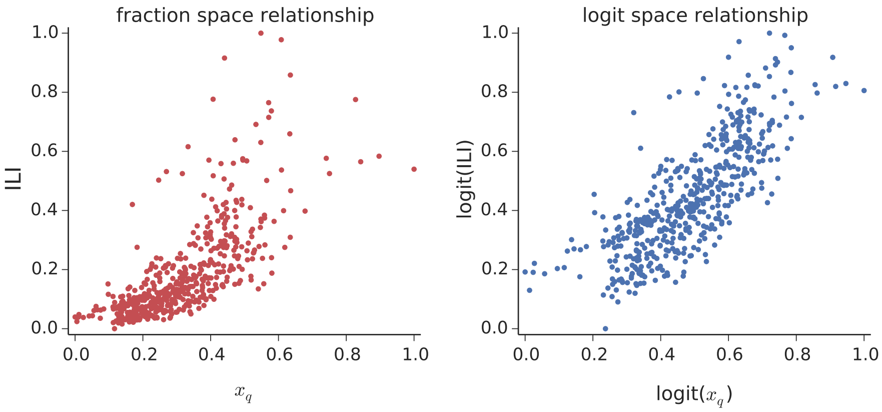

Figure S3. Pairwise relationship of query frequency and ILI rates with (right) and without (left) logit transformation; the query used to draw these plots is ‘dry cough’. Axes have been normalized from 0 to 1.

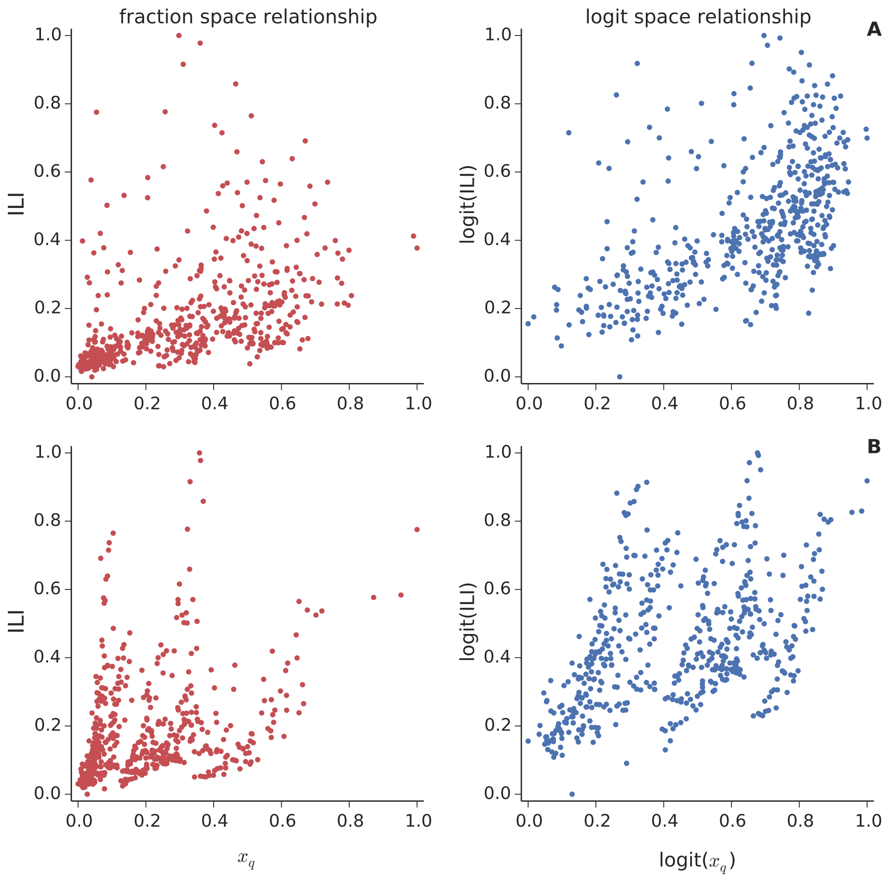

Figure S4. Nonlinearities present in the relationship between ILI and two example queries selected by the Elastic Net with (right) and without (left) the logit transformation. Axes have been normalized from 0 to 1. **A**: 'sex linked traits'. **B**: 'sore throat remedies'.

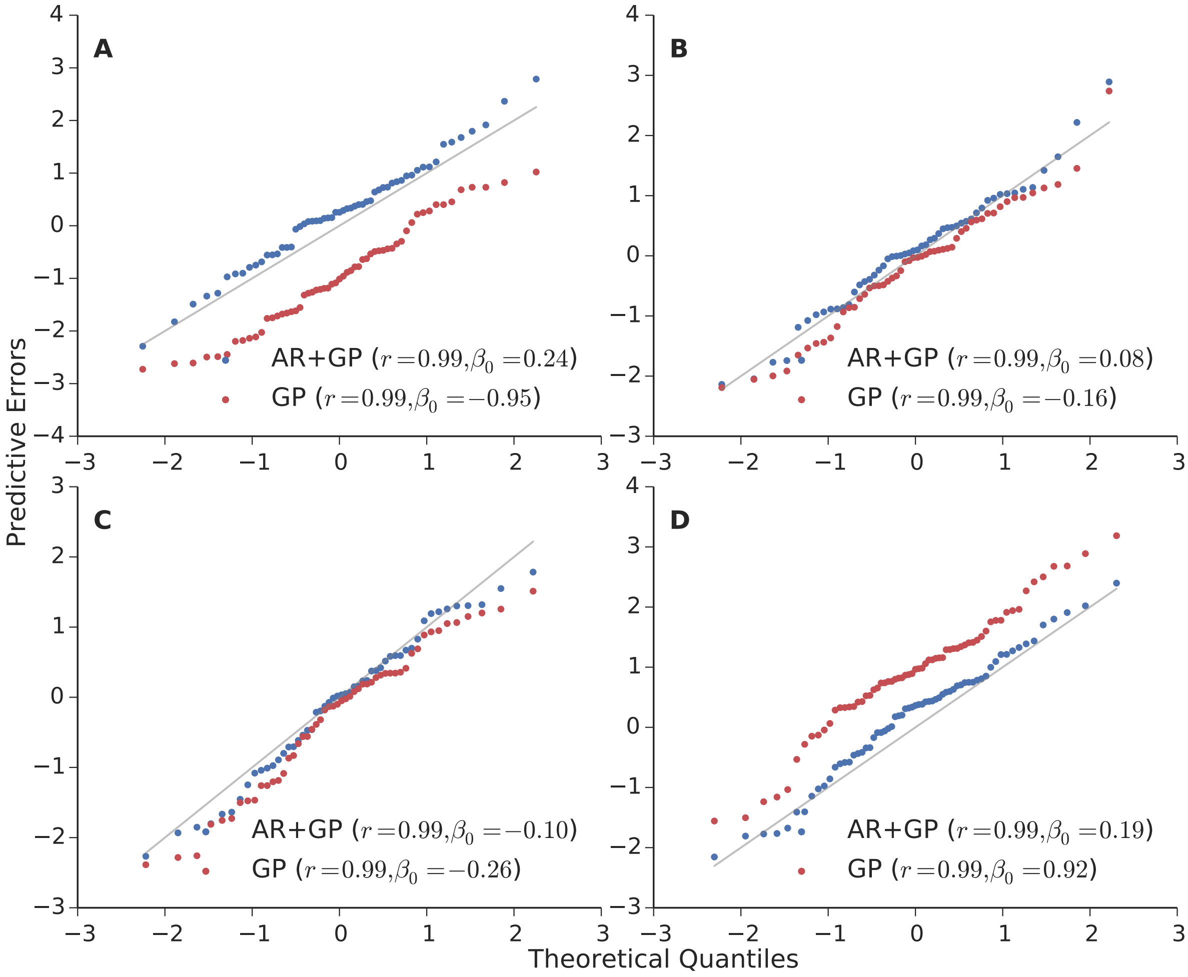

Figure S5. Q–Q plots of rescaled (though not re-centered) predictive errors comparing the GP and AR+GP models in four prediction periods. The addition of the ARMA component dramatically reduces predictive bias. **A – D**: Flu seasons 2009-10, 2010-11, 2011-12 and 2012-13 respectively.

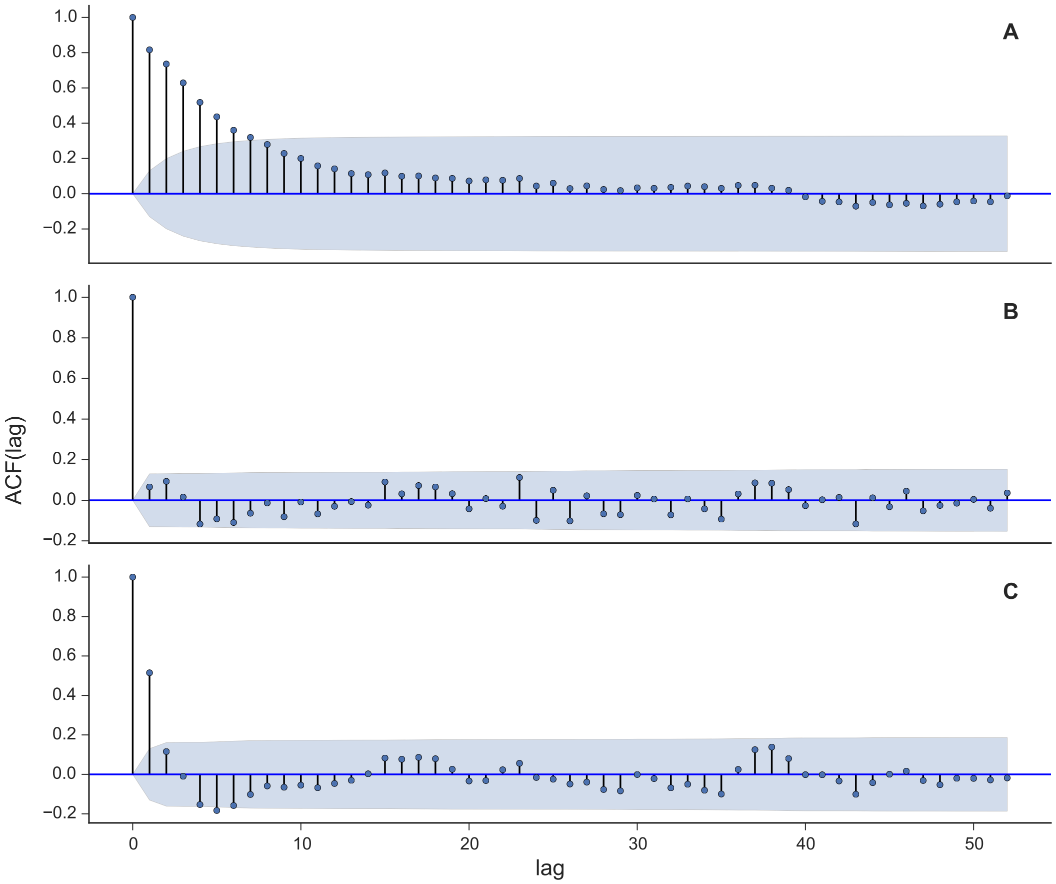

Figure S6. Empirical autocorrelation function of predictive errors. **A**: GP query-only model. **B**: AR+GP model with 1-week lag. **C**: AR+GP model with 2-week lag.

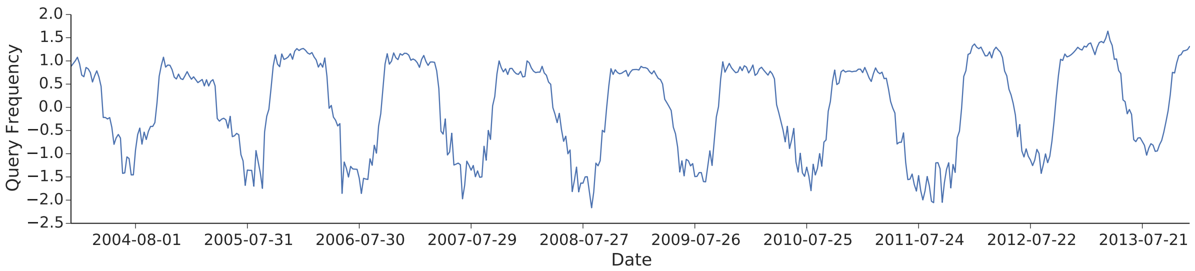

Figure S7. Z-scored, logit-transformed frequency time series for the query ‘nba injury report’. This query is a very good indicator of the winter periods in our data set.

- Testing period 2009–10

```
ARIMA(1,1,1)

Coefficients:
         ar1      ma1  x.train
      0.2510  -0.6856   0.9442
s.e.  0.4097   0.3658   0.0897

sigma^2 estimated as 0.01248:  log likelihood=35.88
AIC=-63.76   AICc=-62.81   BIC=-56.36
```

- Testing period 2010–11

```
ARIMA(2,1,0)

Coefficients:
          ar1      ar2  x.train
      -0.3563  -0.1637   0.9053
s.e.   0.1039   0.0998   0.0533

sigma^2 estimated as 0.008696:  log likelihood=98.64
AIC=-189.27   AICc=-188.87   BIC=-178.69
```

- Testing period 2011–12

```
ARIMA(2,0,2)(1,0,1)[52] with non-zero mean

Coefficients:
         ar1      ar2      ma1     ma2    sar1    sma1  intercept  x.train
      1.5778  -0.6228  -0.8781  0.3034  0.0171  0.3497    -1.3030   0.6968
s.e.  0.0991   0.0956   0.1239  0.0819  0.0614  0.1389     0.2499   0.0557

sigma^2 estimated as 0.004003:  log likelihood=210.6
AIC=-401.7   AICc=-400.47   BIC=-374.19
```

- Testing period 2012–13

```
ARIMA(2,0,2)(1,0,1)[52] with non-zero mean

Coefficients:
         ar1      ar2      ma1     ma2    sar1     sma1  intercept  x.train
      1.7917  -0.8105  -1.1038  0.2529  0.9333  -0.8698    -0.8547   0.7812
s.e.  0.2600   0.2356   0.2540  0.0790  0.4207   0.5286     0.2335   0.0544

sigma^2 estimated as 0.007154:  log likelihood=216.73
AIC=-415.45   AICc=-414.55   BIC=-385.37
```

Figure S8. Output from the `forecast` package,[7] summarizing the final ARIMA fits (with the GP model's predictions as `x.train`). Each prediction period was fit with an increasing amount of data, allowing the procedure to find more complex models in later prediction periods (4 and 5).

- Testing period 2009–10

```
ARIMA(1,0,1) with non-zero mean

Coefficients:
         ar1     ma1  intercept
      0.7700  0.3734    -4.0753
s.e.  0.1091  0.1852     0.1423

sigma^2 estimated as 0.03133:  log likelihood=14.23
AIC=-20.45   AICc=-19.52   BIC=-12.97
```

- Testing period 2010–11

```
ARIMA(0,1,1)

Coefficients:
         ma1
      0.3364
s.e.  0.1026

sigma^2 estimated as 0.0226:  log likelihood=49.43
AIC=-94.86   AICc=-94.74   BIC=-89.57
```

- Testing period 2011–12

```
ARIMA(3,1,2)(1,0,1)[52]

Coefficients:
          ar1      ar2    ar3     ma1     ma2    sar1    sma1
      -0.6251  -0.1262  0.539  1.0646  0.7119  0.1196  0.3129
s.e.   0.1529   0.1264  0.081  0.2078  0.2004  0.0678  0.1693

sigma^2 estimated as 0.009137:  log likelihood=144.89
AIC=-269.72   AICc=-268.74   BIC=-245.32
```

- Testing period 2012–13

```
ARIMA(1,1,1)(1,0,1)[52]

Coefficients:
         ar1      ma1    sar1     sma1
      0.6720  -0.4586  0.8433  -0.6045
s.e.  0.1834   0.2241  0.1795   0.2795

sigma^2 estimated as 0.01389:  log likelihood=142.62
AIC=-275.24   AICc=-274.95   BIC=-258.56
```

Figure S9. Output from the `forecast` package,[7] summarizing the final ARIMA fits (without any query information). Each prediction period was fit with an increasing amount of data, allowing the procedure to find more complex models in later prediction periods (4 and 5).
